# Glassy-like Metal Oxide Particles Embedded on Micrometer Thicker Alginate Films as Promising Wound Healing Nanomaterials

**DOI:** 10.3390/ijms23105585

**Published:** 2022-05-17

**Authors:** Marta Kędzierska, Nisrine Hammi, Joanna Kolodziejczyk-Czepas, Nadia Katir, Maria Bryszewska, Katarzyna Milowska, Abdelkrim El Kadib

**Affiliations:** 1 Department of General Biophysics, Faculty of Biology and Environmental Protection, University of Lodz, Pomorska 141/143, 90-236 Lodz, Poland; maria.bryszewska@biol.uni.lodz.pl (M.B.); katarzyna.milowska@biol.uni.lodz.pl (K.M.); 2 Euromed Research Center, Engineering Division, Euro-Med University of Fes (UEMF), Route de Meknes, Rond-Point de Bensouda, Fès 30070, Morocco; n.hammi@ueuromed.org (N.H.); n.katir@ueuromed.org (N.K.); 3 Department of General Biochemistry, Faculty of Biology and Environmental Protection, University of Lodz, Pomorska 141/143, 90-236 Lodz, Poland; joanna.kolodziejczyk@biol.uni.lodz.pl

**Keywords:** alginate, metal oxide clusters, flexible films, hemolytic activity, prothrombin and thrombine time, wound healing

## Abstract

Micrometer-thicker, biologically responsive nanocomposite films were prepared starting from alginate-metal alkoxide colloidal solution followed by sol-gel chemistry and solvent removal through evaporation-induced assembly. The disclosed approach is straightforward and highly versatile, allowing the entrapment and growth of a set of glassy-like metal oxide within the network of alginate and their shaping as crake-free transparent and flexible films. Immersing these films in aqueous medium triggers alginate solubilization, and affords water-soluble metal oxides wrapped in a biocompatible carbohydrate framework. Biological activity of the nano-composites films was also studied including their hemolytic activity, methemoglobin, prothrombin, and thrombine time. The effect of the films on fibroblasts and keratinocytes of human skin was also investigated with a special emphasis on the role played by the incorporated metal oxide. This comparative study sheds light on the crucial biological response of the ceramic phase embedded inside of the films, with titanium dioxide being the most promising for wound healing purposes.

## 1. Introduction

Polysaccharides are notoriously recognized as biocompatible and abundant building-blocks, built from naturally-designed intriguing architecture units, with additional advantages of featuring functional groups for further modification [1,2]. Commonly, these carbohydrates can be processed in water, under mild conditions, and can be configured in different forms (fibers, films, microspheres, monoliths, fine powders) with possible textural engineering of their secondary structures [3,4,5]. These features have triggered their use as precursors for nanostructured hybrid materials [6,7].

**Figure 1:**
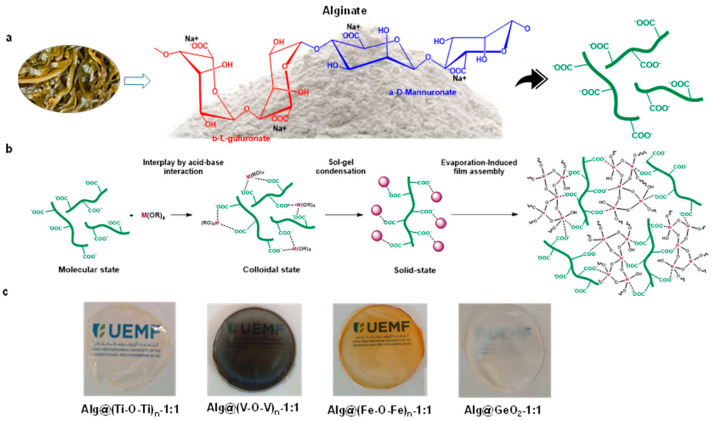
Stepwise preparation of the functional alginate-metal oxide [Alg@(M-O-M)_n_] films. (**a**) chemical structure of alginate. (**b**) a plausible mechanism taking place during the sol-gel process and the film formation. (**c**) digital photos.

Alginate extracted from brown algae is among the most interesting carbohydrates. Its structure consists of alternated blocks of (1,4)-linked urinate residues of pyranose rings featuring carboxylic pendant groups (Figure 1). Alginate is extensively used for the entrapment of biologically active materials for diverse applications such as the controlled release of drug or cosmetics, biological catalysis, food preservation and the transport of enzymes for detergency [8,9]. Alginate also reacts with divalent and multivalent cations to form the so-called ionotropic gels [10]. Alginate can be also distinguished by its outstanding cell encapsulation virtues, compared to its polysaccharide congeners. In essence, the two inherent properties of alginate, namely (i) its ability to form a strong gelling network with tunable strength, and (ii) its stabilizing effect toward metal clusters and nanoparticles, are credited its use to host chemical objects [11,12]. Modified alginate consequently presents enormous potential for further use as wound healing and tissue engineering materials.

Prompted by the possible involvement of its carboxylic acid to stabilize metal alkoxide sol-gel species and the straightforward casting of its body as flexible films, we envisioned that such an approach could led to the preparation of sol-gel modified alginate films. Notably, many nanosized metal oxide particles have been proven to be biologically active, opening indeed a new channel of possibilities in nanomedicine [13,14]. We have consequently investigated different sol-gel precursors to trigger the growth of titanium dioxide, iron oxide, zinc oxide, germanium oxide, and vanadium oxide inside of alginate films. These metal oxides were selected considering their precedents in sol-gel chemistry and the available data concerning their biological activity. Their dissolution further provides an entry to ultra-stable, water-dispersed metal oxide nanoparticles coated on a carbohydrate framework. Biological activity of these films was undertaken to obtain insight into their possible use for tissue engineering and wound healing applications.

## 2. Results and Discussion

### 2.1. Preparation and Characterisation of Alg@(M-O-M)_n_ Films

The preparation of alginate-metal oxide Alg@(M-O-M)_n_ films is a multistep procedure starting from aqueous sodium alginate solution, to which an ethanol solution of metal alkoxide was added dropwise (Figure 1).

After aging and stirring until homogenization, the mixture was poured into a Petri dish and the removal of the solvent triggered sol-gel condensation and growth of the metal oxide clusters and simultaneous densification of the network. The process leads to the formation of micrometer thicker films that can be easily taken off from the initial mold, and handled without precautions. As a result, flexible and transparent crack-free films are obtained as illustrated in Figure 1c. Interestingly, the amount of the metal oxide can be tuned with respect to that of alginate and the resulting films were denoted as Alg@(M-O-M)_n_-X:Y (where Alg refers to alginate, (M-O-M)_n_ refers to the metaloxane network grown inside and X:Y is the amount of alginate and metal oxide engaged. This amount is calculated based on the number of carboxylic groups within the alginate, being 0.231 mmol·g^−1^. As it will be commented later, when the structure of the metaloxane network could be unambiguously established, the expected structure is directly mentioned [ex. GeO_2_ rather that (Ge-O-Ge)_n_] whereas the general formula of (M-O-M)_n_ is used when only the amorphous network was grown inside of the films. We have interestingly noticed that an impressive equimolar amount (1:1) of COO^−^: M could be held within the matrix without disrupting the sol-gel polymerization or the film quality despite the well-established stiffness of the metal oxide phase. This result highlights the importance of the following in-situ synthesis, especially for high metal oxide loading, compared to conventional physical mixing of the formed metal oxide and polymeric alginate that do not provide high-quality films. Above this ratio, the excess of metal oxide with respect to alginate provides less regular films with significant cracking, meaning that there is a limit for the amount of metal oxide to be entrapped while keeping the film quality and transparency.

**Figure 2:**
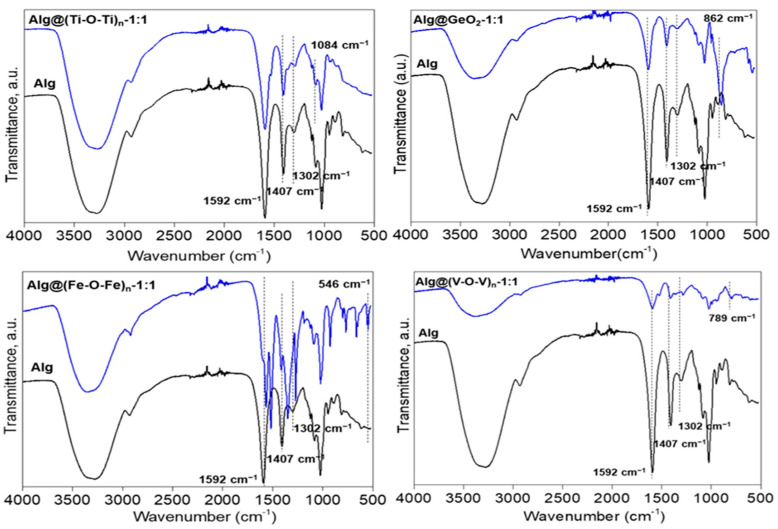
FTIR spectra of Alg and the corresponding Alg@(M-O-M)_n_ nanocomposites.

Observation with the naked eye provides evidence for the incorporation of the metal oxide clusters inside as the film color corresponds to that of the native metal precursor solution (dark green for vanadium oxide, transparent for zinc oxide, light yellow for titanium dioxide and white for germanium oxide) (Figure 1c). The thickness of the films ranges from 15 to 25 µm. EDX mapping was also used to confirm the presence of these inorganic oxide species where the incorporated element was seen in the spectra (Figure S2b). The infrared spectra of the films exhibited asymmetric stretching of the carboxylate groups at 1592 cm^−1^, characteristic of the alginate backbone. Additional typical bands at 1407 cm^−1^ and 1302 cm^−1^ were also observed, indicating the intactness of alginate during the self-assembly and sol-gel polymerization. Besides, the fingerprint of the metal oxide was also noticed at low regions, typical of an extended network of M-O-M based inorganic materials. While those seen for V-O-V at 789 cm^−1^ and for Ti-O-Ti at 1084 cm^−1^ overlap with those of alginate, the ones related to Fe-O-Fe (546 cm^−1^) and Ge-O-Ge (862 cm^−1^) are well distinguished, thereby providing clear evidence for the formation of M-O-M bridges inside of the films (Figure 2 and Figure S3).

**Figure 3:**
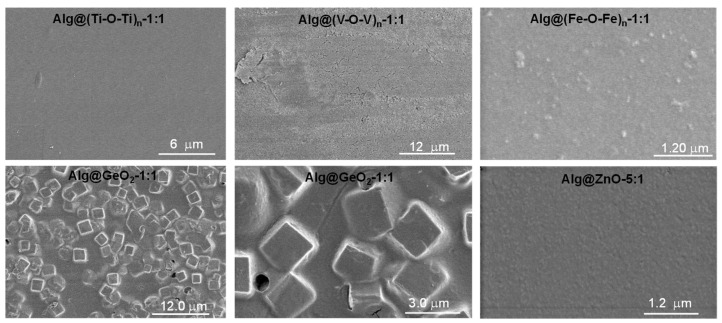
SEM of Alg@(Ti-O-Ti)_n_-1:1, Alg@(V-O-V)_n_-1:1, Alg@(Fe-O-Fe)_n_-1:1, Alg@GeO_2_-1:1 and Alg@ZnO-5:1 films.

SEM analyses of Alg@(Ti-O-Ti)_n_-1:1 show a smooth surface with no visible clustering or aggregation that may be assigned to uncontrolled growth of the metal oxide species. In the case of Alg@ZnO-1:1, regular discrete nanoparticles are visible and in the case of Alg@(Fe-O-Fe)_n_-1:1 slightly bigger objects can be distinguished from the network, as a result of the significant growth of the resulting metal oxide particles (Figure 3 and Figure S2a). However, the pattern is very typical of well-controlled nanoparticles rather that randomly grown surfactant-free nanoparticles. Nice regular nano-squares are also observed in the case of Alg@GeO_2_-1:1 where the squares are attributed to the formation of crystalline germanium oxide. However, the flexibility of the film is also maintained as it is for other materials, meaning that the growth of the crystalline phase has occurred intimately within the alginate network, thereby excluding the phase separation (Figure 3).

X-ray diffraction (XRD) reveals that, depending on the metal precursor, amorphous or crystalline phase could be formed inside (Figure S4). Indeed, no crystalline peaks could be observed in the case of Alg@(Ti-O-Ti)_n_-1:1, Alg@ZnO-5:1 and for Alg@ZnO(Cl)-5:1 meaning that only amorphous titanium dioxide and zinc oxide could be formed under our conditions. Some crystalline peaks were observed in the case of Alg@(V-O-V)_n_-1:1. Well-resolved crystals are also noticed in the case of Alg@GeO_2_-1:1, that match perfectly with α-quartz-like trigonal GeO_2_ structure with P3221 hexagonal symmetry. The size of the crystal is of 51 nm as estimated by Scherrer equation. In the case of Alg@(Fe-O-Fe)_n_-1:1, XRD reveals the presence of one diffraction peak at 2θ value of 13°. This single crystal fits well with the crystalline structure of Fe_3_(CO)_12_ with an average size of 9.73 nm. These similarities suggest the formation of three iron clusters ligated by carboxylic groups following the well-known egg-model and their confinement inside.

High thermal stability was noticed for alginate-metal oxides Alg@(M-O-M)_n_ film as compared to the native alginate film owing to the successful incorporation of metal oxide within the film (Figure S5). A significant increase of T50 (50 wt% decomposition temperature) was observed from 271 °C for alginate film to 495 °C for Alg@(Ti-O-Ti)_n_-1:1, with an impressive ∆T of 224 °C. Furthermore, up to 28 wt% of inorganic oxide residue remained after complete degradation of the polysaccharide films at 900 °C.

**Figure 4:**
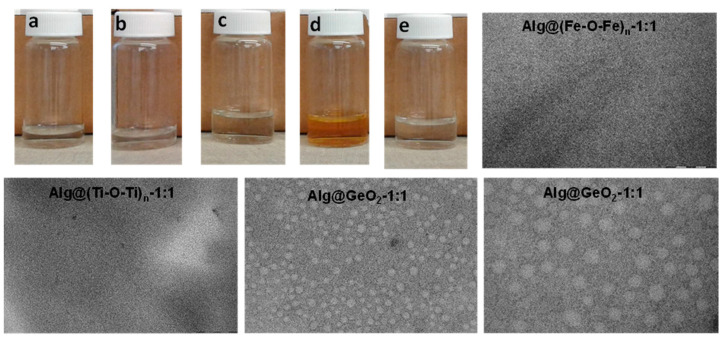
Digital photos and TEM. (**a**) Alg. (**b**) Alg@(Ti-O-Ti)_n_-1:1, (**c**) Alg@(V-O-V)_n_-1:1, (**d**) Alg@(Fe-O-Fe)_n_-1:1, (**e**) Alg@GeO_2_-1:1 and their corresponding transmission electronic microscopy.

We have next triggered disintegration of alginate-based hybrid films under aqueous conditions (Table S1b). Alg-(M-O-M)_n_ could be dissolved easily in neutral solutions compared to analogues chitosan-based films that necessitate gentle acidic solution [15]. Alg@GeO_2_-1:1, Alg@(Fe-O-Fe)_n_-1:1 and Alg@(V-O-V)_n_-1:1 dissolve instantaneously to give a long term stable dispersed solution (Figure 4a–e). Visualization of these solutions by transmission electron microscopy confirms the formation of discrete and small nanoparticles (Figure 4 and Figure S6). The smallest size (nearly 5 nm) observed in the case of Alg@(Fe-O-Fe)_n_-1:1 and Alg@(Ti-O-Ti) _n_-1:1, which is the consequence of the structure-directing growth exerted by alginate, is consistent with the amorphous nature of the metal oxide phase, as a substantial expansion is necessary to trigger crystallization. In the case of germanium oxide, regular spherical particles of nearly 80 nm are observed upon dissolution, meaning that germanium oxide crystals are built from the association of these elementary nanoparticles. More interestingly, these solutions maintain their colloidal stability for an extended time of two months, with no noticeable evolution or sedimentation. This long-term stability contrasts with the high surface energy of metal oxide particles in water, and could be explained by an intimate interplay of the metal oxide and the alginate backbone where the latter ensure favorable interfacial interactions with the milieu while preventing the coarsening of the metal oxide nanoparticles. A similar behavior was previously disclosed for water-dispersed Prussian blue nanoparticles prepared by dissolution of porous polysaccharide-Prussian blue aerogels [16] and by dismantling of chitosan-mixed metal oxide films [15]. To sum up, the prolonged stability of these colloidal solutions is due to the presence of metal oxide nanoparticles (with a small size) and their strong interplay through hydrogen-bonding with alginate.

### 2.2. Hemolytic Activity and Hb Adsorption

**Figure 5:**
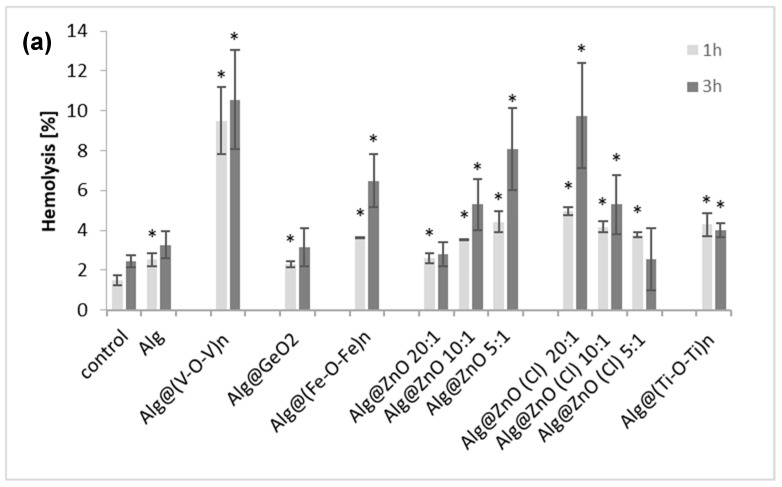

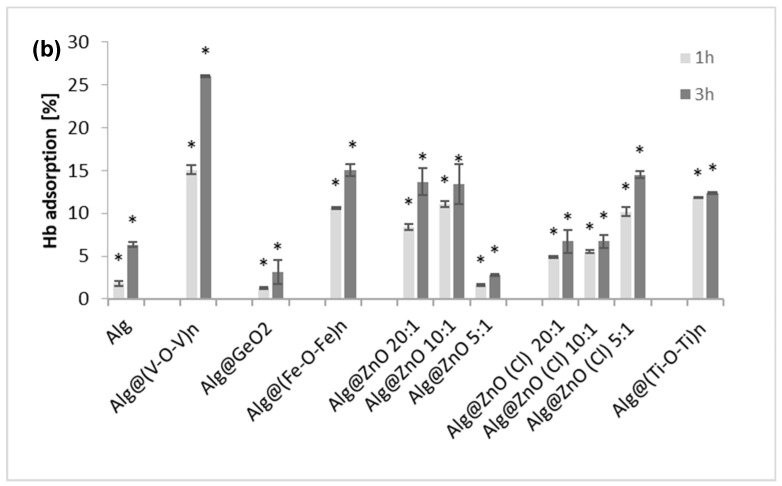
(**a**) Hemolysis of erythrocytes incubated with alginate-metal oxide nanocomposite films for 1 and 3 h. All changes are statistically significant compared with the control (*n* = 6, * *p* < 0.05). (**b**) Hemoglobin adsorption by different alginate-metal oxide composites incubated for 1 and 3 h (*n* = 10, * *p* < 0.05).

Human erythrocytes hemolysis was determined by measuring the hemoglobin content after incubation (1 and 3 h) with alginate nanocomposites (Figure 5a). We observed that all alginate-metal oxide nanocomposites induced hemolysis. The degree of hemolysis depends on the metal oxide entrapped inside and the incubation time. For 1-h incubation, the largest hemolysis of 9% was caused by Alg@(V-O-V)_n_-1:1 while the lowest value (2%) was for Alg@GeO_2_-1:1. For the other films, the hemolysis ranged from 2.5 to 5% and increases to around 3–11%. after 3 h of incubation. Again, the highest hemolysis was induced by Alg@(V-O-V)_n_ film and the lowest hemolysis was observed for Alg@ZnO(Cl)-5:1 and Alg@ZnO-20:1. Notably, the percentage of hemolysis measured for Alg@ZnO(Cl)-5:1 and Alg@(Ti-O-Ti)_n_-1:1 was higher after 1 h of incubation than after 3 h of incubation.

The decrease in hemolysis after prolonged incubation gave grounds to check the adsorption of hemoglobin on the surface of the films (Figure 5b). As in hemolysis, adsorption of hemoglobin depended on the type of their modification. Adsorption ranges from 1.5% to 15% after 1 h of incubation and from 3% to 26% after 3 h. The lowest percentage of adsorption after 3 h of incubation was recorded for Alg@ZnO-5:1 and Alg@GeO_2_-1:1 films, and the highest for Alg@(V-O-V)_n_-1:1. The results suggest that Hb released from erythrocytes is adsorbed to alginate materials, which results in a decrease in the hemoglobin content in the solution, which was regarded as no increase in hemolysis after 3 h of incubation. A low hemolysis and adsorption of Hb after incubation with alginate alone film (Alg) and a germanium oxide-containing film (Alg@GeO_2_-1:1) may suggest that these two films are the least hemotoxic. These results are in agreement with the previously reported literature. The highest hemolysis was observed for Alg@(V-O-V)_n_-1:1. Precedents have shown that vanadium pentoxide induces 65% hemolysis of red blood cells [17] while other works have suggested the involvement of vanadium species in producing lipid peroxidation in the erythrocyte membrane, leading to hemolysis, with a possible interference with the erythroid differentiation process [18]. A correlation between the degree of hemolysis and the decrease in the reduction of peripheral erythrocytes after exposure to vanadium has been also demonstrated [19]. For Alg@ZnO films, hemolysis was directly proportional to the concentration of ZnO in the films, in agreement with the literature [20,21]. An opposite trend was however observed for Alg@ZnO(Cl), which points to the role of the starting precursor that contain chloride. However, adsorption of hemoglobin may interfere with the determination of the level of hemolysis.

### 2.3. Methemoglobin

We next turned our attention to measure the percentage of methemoglobin. In fact, circulating hemoglobin is always at risk of being oxidized to methemoglobin, in which the molecule retains its original tetrameric structure but can no longer carry oxygen.

**Figure 6:**
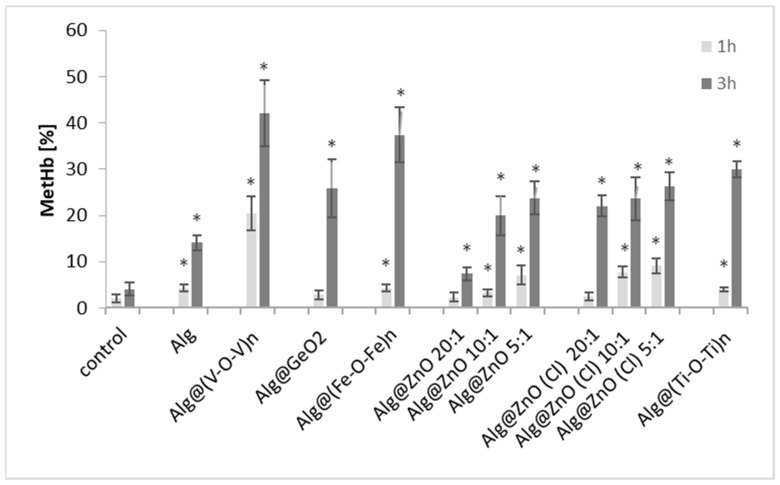
Percentage of methemoglobin in erythrocytes incubated with alginate composites for 1 and 3 h. All changes are statistically significant compared with the control (*n* = 6, * *p* < 0.05).

We herein noticed that most alginate-metal oxide films cause oxidation of hemoglobin after 1 h incubation with erythrocytes (Figure 6). The measured methemoglobin was only 2% for the control after 1 h of incubation and increases to 4% after 3 h. After 1 h incubation with alginate films, the highest methemoglobin content (20%) was in the sample incubated with Alg@(V-O-V)_n_-1:1. Statistically significant changes in the percentage of met-Hb content were observed for all alginate composites after 3 h incubation. A percentage of 7% was measured for Alg@ZnO-20:1 while a high amount of met-Hb was rather measured for Alg@(Fe-O-Fe)_n_-1:1 (37%) and for Alg@(V-O-V)_n_-1:1 (42%). We indeed conclude that the presence of iron oxide and vanadium oxide in alginate films contributes to the increase in the level of metHb in erythrocytes in in vitro tests.

### 2.4. Cytotoxicity (MTT)

**Figure 7:**
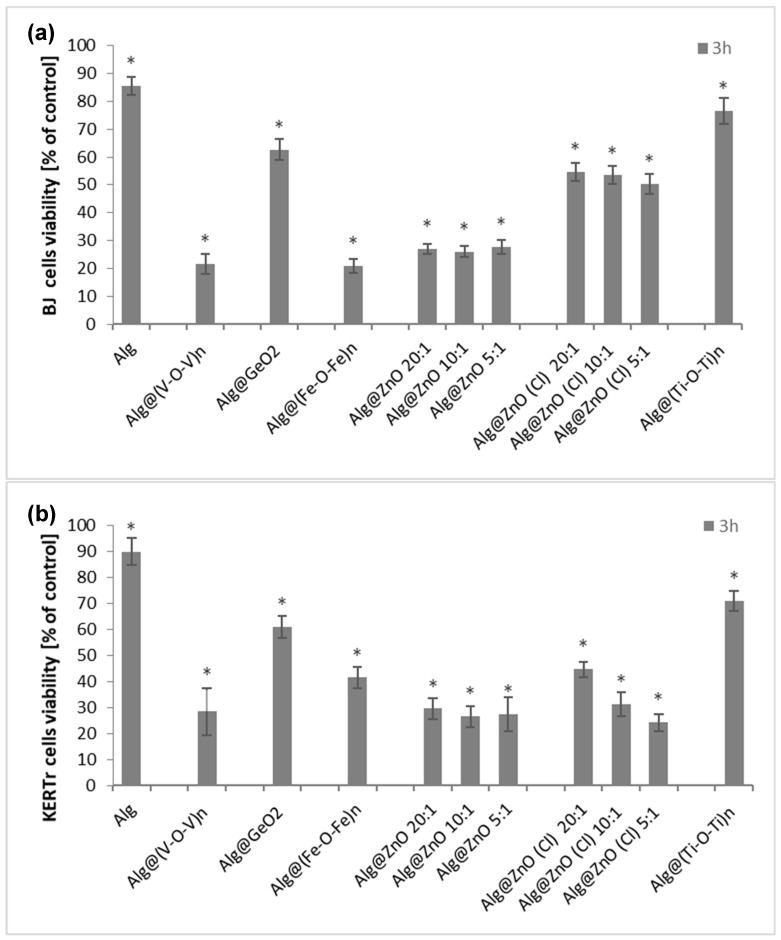
Viability of BJ (**a**) and KERTr (**b**) cells treated with alginate composites for 3 h. All changes are statistically significant compared with the control (*n* = 6. * *p* < 0.05).

In order to assess the cytotoxicity of the tested composites on fibroblasts and keratinocytes of human skin, the MTT test was performed. Cytotoxicity of biocomposites was assessed based on cell viability after 3 h of incubation (Figure 7). The percentage of viable cells was calculated relative to controls (cells incubated without biocomposite) whose viability was considered 100%. All the test nanocomposites decreased the viability of BJ and KERTr cells, but unmodified alginate film can be considered non-toxic as cell viability is above 80%. After incubation of cells with Alg, BJ cell viability was around 85% and KERTr around 90%. The Alg@(Ti-O-Ti)_n_ film also showed low toxicity in consistency with the literature. Kecelil et al. [22] showed that titanium oxide did not cause a large decrease in the viability of fibroblasts, the viability of fibroblasts incubated with TiO_2_ was comparable to the control. The most toxic composites to fibroblasts were Alg@(V-O-V)_n_ and Alg@(Fe-O-Fe)_n_, reducing the cells viability to ~20% (Figure 7a). For keratinocytes, zinc-containing composites were the most toxic, with Alg@ZnO(Cl)-5:1 recording only 24% viability and 27% for Alg@ZnO-10:1 (Figure 7b). The obtained results showed indeed that the cytotoxicity of the films is caused by the presence of metal oxides and varies depending on their type.

These results are not surprising given the well-established toxicity of metal oxide nanoparticles, which are mainly attributed to the adsorption of protein on their highly-reactive surface. The adsorption capacity of metal oxide nanoparticles is an important factor in the in-vitro cytotoxicity assessment for low toxicity materials [23,24,25]. Besides, it has been also mentioned that some metal oxide nanoparticles act as a reservoir to release soluble substances that possibly induce severe mitochondrial dysfunction and induction of intracellular ROS [24]. Given that alginate-metal oxide films behave by themselves as precursors for soluble nanoparticles, this could explain the reduced viability noticed with these films.

### 2.5. ROS Generation

**Figure 8:**
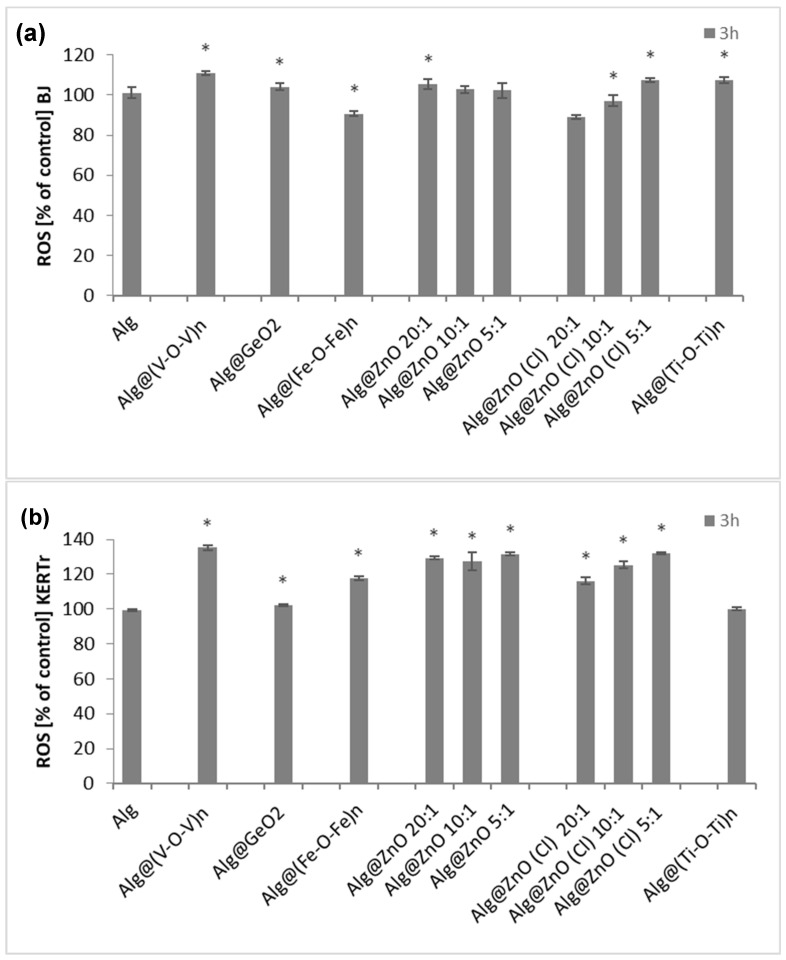
Percent of reactive oxygen species in BJ (**a**) and KERTr (**b**) cells incubated by 3 h with alginate composites. (*n* = 6, * *p* < 0.05).

For the determination of the role of reactive oxygen species (ROS) in the cytotoxic activity of alginate biocomposites, BJ and KERTr cells were incubated with the tested materials for 3 h. The results were referenced to a 100% control. Alginate films did not induce ROS, but alginate-metal oxide films slightly induced oxidative stress, leading to an increase in the number of intracellular reactive oxygen species (Figure 8). The level of ROS was the highest for both cell lines for Alg@(V-O-V)n-1:1 and was about 110% for BJ and 135% for KERTr. For composites Alg@ZnO-5:1, Alg@ZnO (Cl)-5:1 and Alg@(Ti-O-Ti)_n_-1:1 ROS level in fibroblasts was in the range of 105–107%, while Alg@(Fe-O-Fe)_n_-1:1, Alg@ZnO(Cl)-20:1, Alg@ZnO(Cl)-10:1 did not generate ROS. Their level was lower or the same as in the control. However, the level of reactive oxygen species in keratinocytes after incubation with the modified films was in the range of 105–135%. Only Alg@(Ti-O-Ti)_n_-1:1 did not contribute to the increase in ROS levels.

It is well known that metal oxide nanoparticles induce oxidative stress in culture cells [26]. In A549 cells, remarkable increase of the intracellular ROS level was induced by ZnO and ZnO-S exposure [27]. Moreover, internalized nanoparticles produce ROS directly and indirectly via mitochondrial dysfunction [26]. However, ROS generated by nanoparticles metal oxides play signaling and regulatory roles (antiseptic role in wound healing and wound-to-leucocyte signaling) in tissue engineering [28,29,30]. Metal oxide nanoparticles show indeed good potential for therapy of different diseases and pathogen control. Aside from the important biological functions of ROS such as cell proliferation, migration, signalization, wound healing and so forth, the generated reactive species could have a detrimental effect above a certain amount by producing an inflammatory response and generating oxidative stress and eventual apoptosis of normal cells [31].

### 2.6. Assessment of Mitochondrial Membrane Potential (ΔΨm)

**Figure 9:**
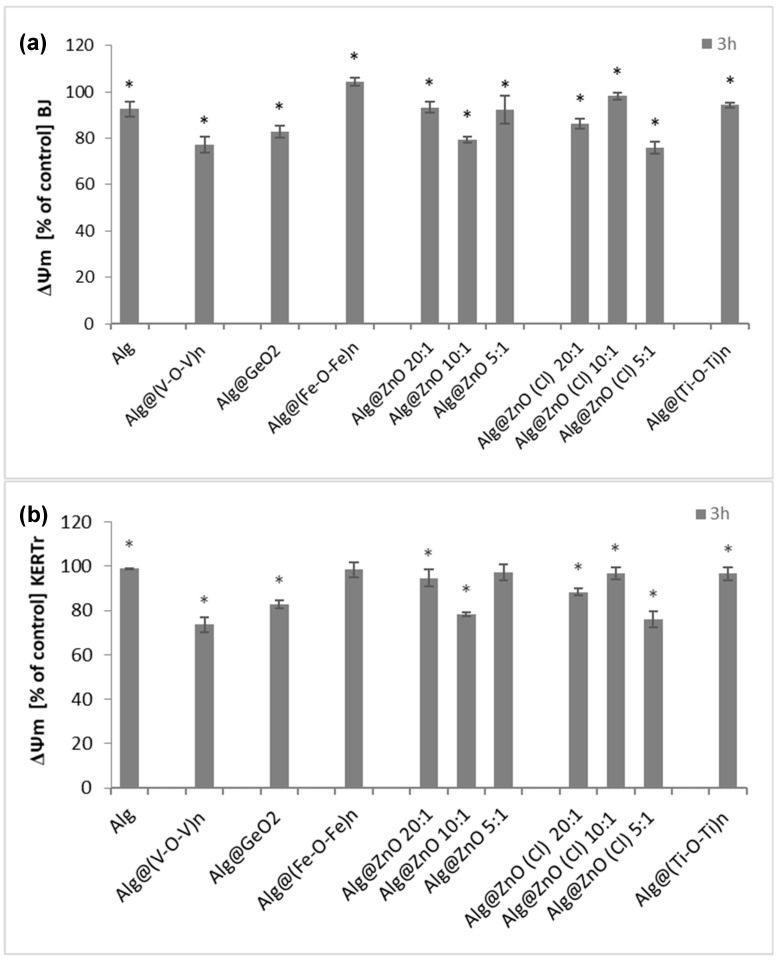
Changes in mitochondrial membrane potential (ΔΨm) after incubation (3 h) with alginate composites in (**a**) BJ and (**b**) KERTr cells (*n* = 6, * *p* < 0.05).

The method using the JC-1 probe allows to assess the proapoptotic activity of alginate biomaterials modified with metal oxides, associated with the change of transmembrane mitochondrial potential (ΔΨm). Our studies have shown that most biocomposites exhibit low proapoptotic capacity (Figure 9). Incubation of BJ cells with alginate films caused slight depolarization or hyperpolarization of the mitochondrial membrane (Figure 9a).

The largest decrease in ΔΨm was observed for Alg@ZnO(Cl)-5:1, Alg@(V-O-V)_n_ or Alg@ZnO-10:1, while there was an increase for Alg@(Fe-O-Fe)_n_. Similar results can be observed in the case of KERTr keratinocytes incubated for 3 h with biocomposites. The greatest decrease in mitochondrial potential is seen in cells incubated with Alg@ZnO-10:1, Alg@ZnO(Cl)-5:1 and Alg@(V-O-V)_n_. The remaining composites did not cause significant changes in potential and remained at the control level. These studies suggest that it is the type of metal oxide modifying the alginate composite that influences the changes in the mitochondrial potential. Previous studies have demonstrated that alginate does not cause changes in mitochondrial activity in fibroblasts (NIH 3T3) [32,33] while other works have gained insight into the role of zinc oxide nanoparticles for the reduction of mitochondrial membrane potential [34]. A substantial decrease in mitochondrial potential was also associated to the presence of vanadium oxide, which consolidate the results obtained herein with Alg@(V-O-V)_n_ [35,36,37,38,39].

### 2.7. Genotoxicity (Comet Assay)

**Figure 10:**
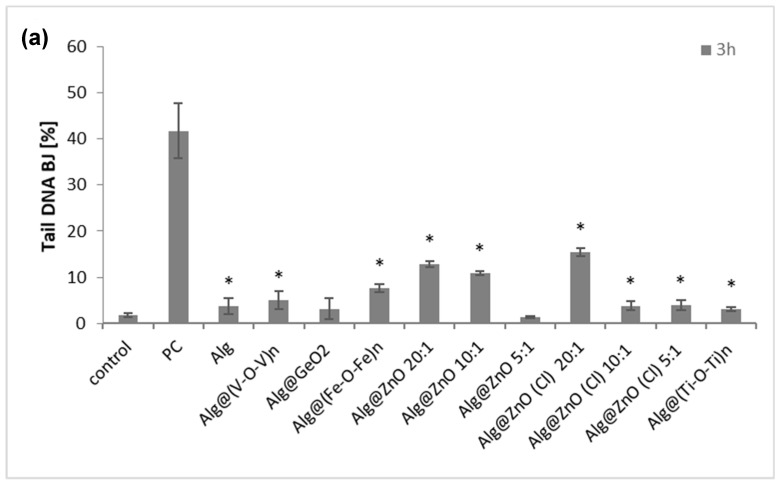

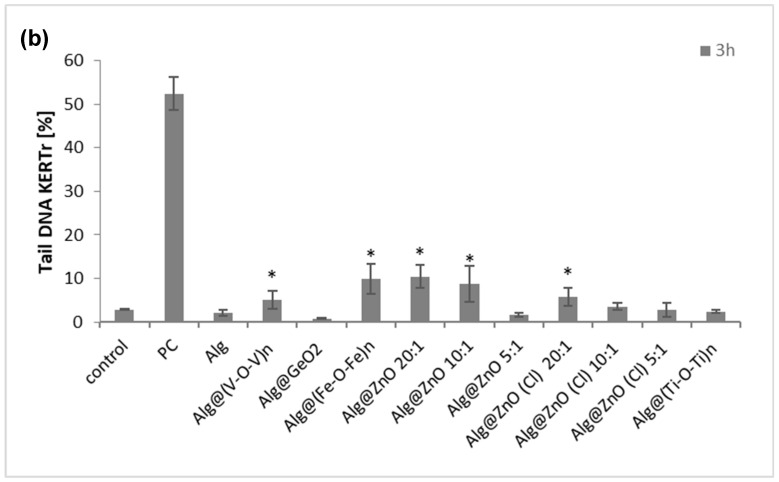
The genotoxicity of alginate composites to BJ cells (**a**) and KERTr cells (**b**) incubated for 3 h (PC- positive control; cells treated with H_2_O_2_), *n* = 6. * *p* < 0.05.

An important aspect of testing the toxicity of any material intended to use in wound healing is the determination of their genotoxicity. The influence of all types of composites on the DNA of skin cells is an extremely important stage in in vitro research. Comet assay identifies single-stranded and double-stranded DNA breaks as well as any chemical and enzymatic modifications that can turn into DNA breaks or chromatids. The results presented in Figure 10 show the comet tail length as a percentage of the level of damage. After 3 h of incubation, the percentage of the tail for BJ and KERTr amounted to a maximum of 10–15%. For fibroblasts the most genotoxic nanocomposites were Alg@ZnO(Cl)-20:1, Alg@ZnO-20:1, Alg@ZnO-10:1, while Alg@(Fe-O-Fe)_n_ can be ranked as slightly less toxic. For keratinocytes the strongest DNA damaging effect was noticed for Alg@(Fe-O-Fe)_n_, Alg@ZnO-20:1, Alg@ZnO-10:1. Indeed, the entrapment of iron oxide and zinc oxide instead of the alginate films induces a significant damage in DNA, whereas no damage can be associated to the use of native alginate Alg, as the tail length percentage was very low, about 4% for fibroblasts and 2% for keratinocytes. A critical survey of the literature shows the genotoxicity of titanium dioxide, zinc oxide, iron oxide and most of them occur through DNA oxidation by ROS and DNA strand breaks [40,41,42,43,44,45,46,47].

### 2.8. Migration Cells Fibroblasts and Kerationcytes

**Figure 11:**
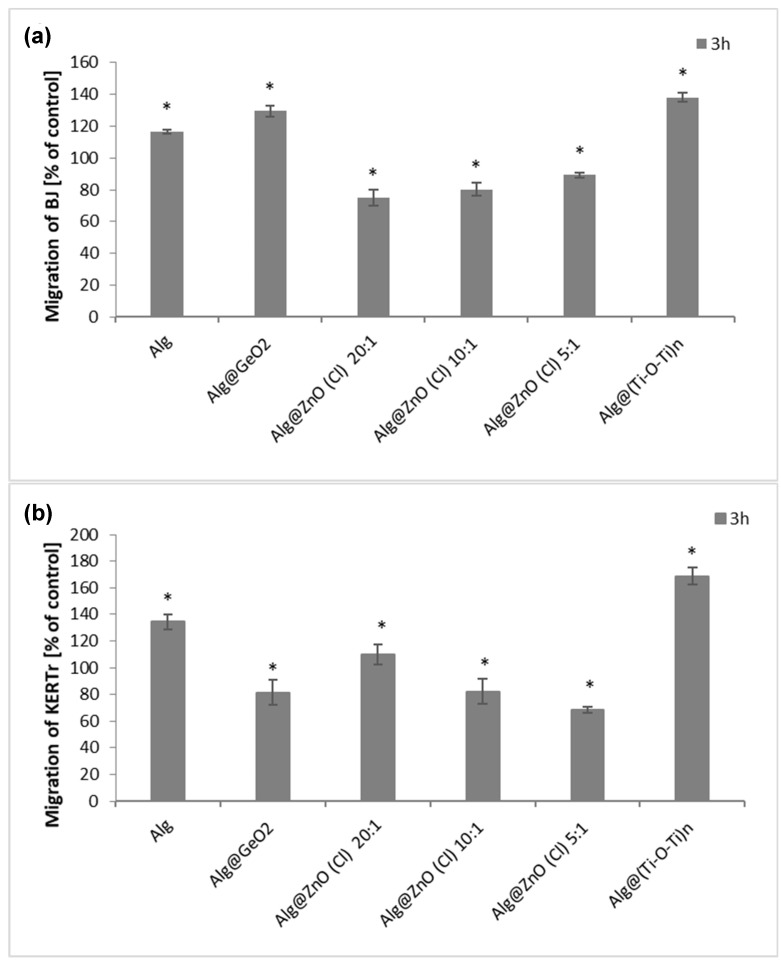
The migration of (**a**) BJ, (**b**) KERTr cells incubated for 3 h with modified alginate biocomposites (*n* = 6. * *p* < 0.05).

The skin cell migration process plays an important role in closing a wound and rebuilding damaged tissue. To assess the influence of alginate nanocomposites on the migration process of BJ and KERTr cells, we have selected films displaying lowest toxicity against the cells. We noticed that some of the tested composites increased the migration of both fibroblasts and keratinocytes after 3 h of incubation (Figure 11). Alginate alone (Alg) increased the migration of both type of cells. Alg@(Ti-O-Ti)_n_ had the greatest impact on the stimulation of the migration of fibroblasts and keratinocytes, for which the migration level was about 140% for BJ and about 170% for KERTr. Alg@GeO_2_ composite also activated the migration of fibroblasts, but slightly reduced the migration of keratinocytes. On the other hand, all selected alginates composites containing zinc oxide (Alg@ZnO(Cl)) caused a reduction in cell migration compared to the control, which might be due to the toxicity of Alg@ZnO(Cl). To summarize, Alg, Alg@(Ti-O-Ti)_n_ and Alg@GeO_2_ stimulate the migration of skin cells, which may potentially affect faster wound healing. These results complement those previously reported in the literature on the positive effect of alginate-based materials for cell migration and explain the extensive use of alginate derivatives in tissue engineering [48,49,50,51,52].

### 2.9. Prothrombin Time (PT), Thrombin Time (TT) and Amidolytic Activity of Thrombin

**Figure 12:**
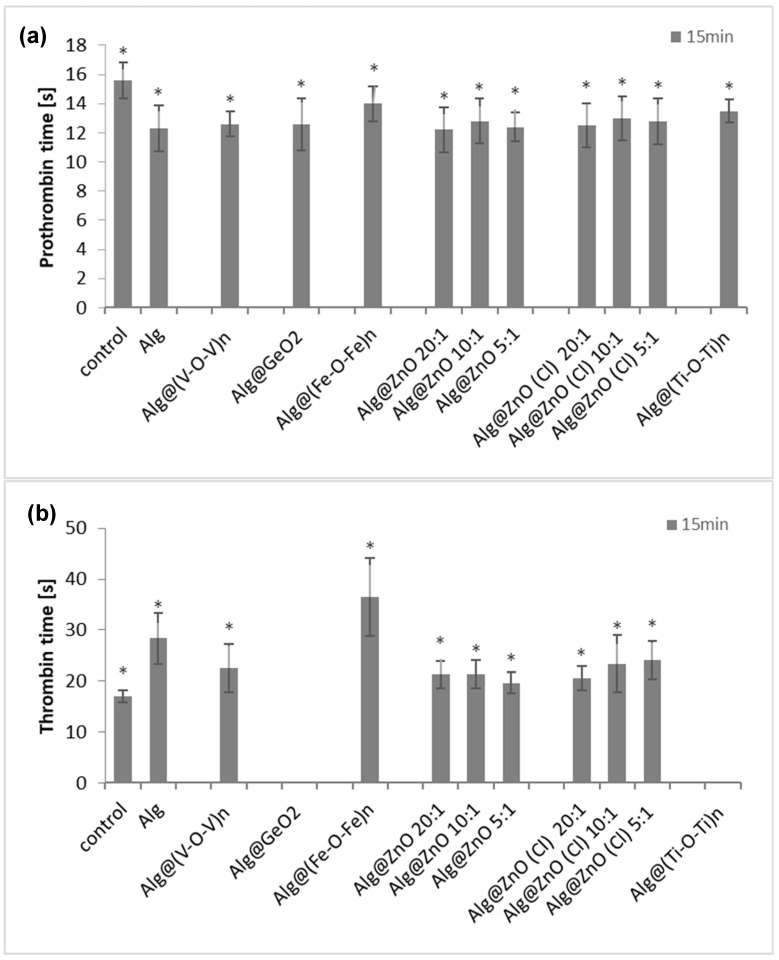

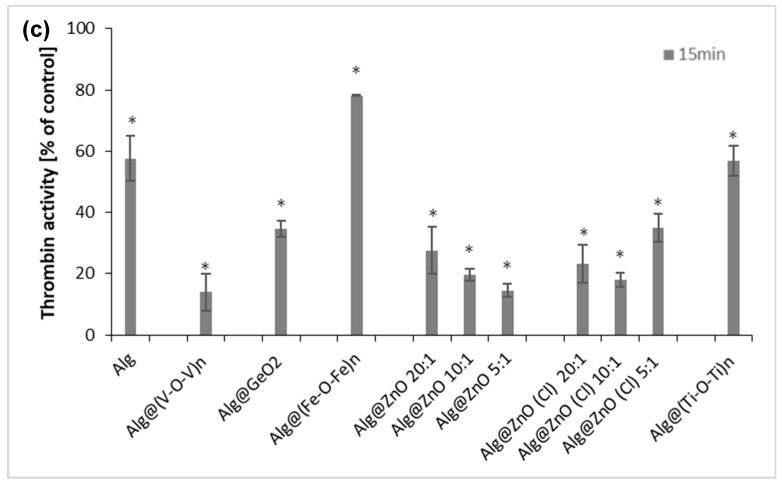
Effects of the examined alginate composites on human blood plasma clotting parameters: (**a**) Prothrombin time in plasma incubated with alginate composites. All changes are statistically significant compared with the control (untreated plasma) (*n* = 8, * *p* < 0.05); (**b**) Thrombin time in plasma incubated with alginate composites. All changes are statistically significant compared with the control (untreated plasma) (*n* = 8, * *p* < 0.05); (**c**) Amidolytic activity of thrombin incubated with alginate composites. All changes are statistically significant compared with the control (*n* = 4, * *p* < 0.05). In the thrombin amidolytic assay, the activity of control (untreated enzyme) was assumed as 100%.

To determine the effects of the examined composites on the hemostatic properties of blood plasma, two types of blood clotting tests, i.e., the prothrombin time (PT) and the thrombin time (TT), were used. The PT parameter reflects the tissue factor-induced activation of the blood plasma coagulation cascade, and provides information on functionality of the extrinsic pathway of coagulation and thrombin generation. Figure 12a shows that the prothrombin time (PT) was slightly reduced in plasma containing the functionalized alginate films compared to the control. The PT for control was 15.6 s. The shortest time was obtained after incubation with Alg@ZnO-20:1 and it was 12.2 s. The weakest effect on reducing the clotting time was observed for Alg@(Fe-O-Fe)_n_-1:1 and was equal to 14 s. Measurements of the prothrombin time demonstrated indeed that alginate-based nanocomposites shortened this clotting time, i.e., increased the activation of prothrombin and generation of the thrombin enzyme

The thrombin time corresponds to the last step of blood clotting, during which fibrinogen is transformed into fibrin by thrombin. Surprisingly, in the TT test, the anti-coagulant effects was found. Figure 12b. shows that the thrombin times were prolonged in plasma incubated with alginate films, when compared to the control. Incubation of plasma with alginate films had no protective effect on plasma clotting ability. For the control, the thrombin time was about 18 s. Alg@(Fe-O-Fe)_n_-1:1 had the strongest effect on extending of thrombin time—it was about 36 s, being twice as long as in control. Films: Alg@GeO_2_-1:1 and Alg@(Ti-O-Ti)_n_-1:1 did not show procoagulant abilities.

Our findings indicated modulatory effects of the examined alginate nanocomposites and their diverging actions at different steps on the blood plasma coagulation cascade. While the coagulation cascade activation and propagation were stimulated by the examined alginate nanocomposites (acc. to PT results), at the level of the active enzyme (thrombin), these substances acted rather as inhibitors of coagulation (TT results). Thus, to determine whether the examined composites were able to inhibit the thrombin activity, additional experiments devoted to their influence on this enzyme were also performed. Results from the amidolytic activity of thrombin exposed to the alginate composites evidently demonstrated their thrombin-inhibitory effects (Figure 12c). The most effective thrombin inhibitors were Alg@ZnO-10:1, Alg@ZnO-5:1 and Alg@ZnO(Cl)-10:1, reducing the hydrolytic activity of this enzyme by about 80%. Mechanistically speaking, the blood coagulation process is a complex cascade of enzymatic reactions associated with an extensive network of other interactions, including cofactor properties of the coagulation surface, allosteric modulation of enzyme activities as well as both the positive and negative feedback effects [53]. Thrombin enzyme is susceptible to regulation by different factors, including ions, allosteric modulators and direct or indirect inhibitors [54]. Its structure contains two anion-binding exosites (I and II), responsible for interactions with negatively charged regions on cofactors and other molecular targets for thrombin [55]. Herein, measurements of the PT and the thrombin amidolytic activity assay demonstrated that the alginate nanocomposites may act as thrombin inhibitors. Considering the negatively charged surface of alginate nanocomposites, their interactions with the exosites I and II seem to be the most likely mechanisms of allosteric regulation of this enzyme by the presence of these materials and their inhibitory action on thrombin.

## 3. Materials and Methods

### 3.1. Chemical and Reagents

Titanium diisopropoxide bis (acetylacetonate) (Ti(acac)_2_OiPr_2_), Vanadium III acetylacetonate (V(acac)_3_), iron (III) acetylacetonate (Fe(acac)_3_), Zinc acetate (Zn(OAc)_2_), Zinc chloride (ZnCl_2_), Germanium(IV) ethoxide (Ge(OEt)_4_), absolute ethanol, were obtained from Sigma Aldrich (Hamburg, Germany), and used without any further purification. Sodium alginate was supplied by Sigma Aldrich, and used as received.

### 3.2. Characterisation Techniques

Fourier transformed infrared spectra were obtained with a PerkinElmer Spectrum 100FT- IR spectrometer on neat samples (ATR FT-IR) (PerkinEmler, Shelton, CT, USA). X-ray powder diffraction (XRD) patterns were recorded on a D8 Advance Bruker AXS system (Karlsruhe, Germany) using Cu Kα radiation with a step size of 0.02° in the 2θ range from 10° to 80° for WAXS (geometry: Bragg–Brentano, θ/2θ mode). Scanning electronic microscopy (SEM) images were obtained using a JEOL JSM 6700F (SEMTech Solution, North Billerica, MA, USA). Transmission electronic microscopy (TEM) images were obtained using JEOL (JEOL JEM2010, 200 Kv, Pleasanton, CA, USA) at an activation voltage of 200 kV. Thermogravimetric analyses (TGA) were obtained with a thermal analyzer instrument Q500 (TA Instruments, New Castle, DE, USA) on the range 25–1000 °C at heating speed of 5 °C/min.

### 3.3. Preparation of Alginate-Metal Oxide Films

A quantity of 0.05 g of sodium alginate powder (0.231 mmol of carboxylic acid sodium salt unit) was dissolved in 4 mL of deionized water and stirred for 1 h. Next, a selected ethanol solution of metal precursor [Ti(acac)_2_OiPr_2_, Zn(OAc)_2_, ZnCl_2_, Ge(OEt)_4_ and Fe(acac)_3_] with different molar ratio with respect to COO^−^ unit of alginate, was added to the transparent solution. The mixture was stirred for additional 2 h at room temperature until complete homogenization. The resulting solution was cast onto a clean petri dish at room temperature for 24 h until total evaporation of the solvent. Specific experimental conditions are given in Table S1a.

### 3.4. Materials for Biological Studies

Human fibroblast BJ (CRL-2522) and human keratinocyte CCD 1102 KERTr (CRL2310) cell line were purchased from American Type Culture Collection ATCC^®^ (Manassas, VA, USA). Keratinocyte serum-free medium with added keratinocyte supplements, including bovine pituitary extract (BPE), human recombinant epidermal growth factor (EGF), fetal bovine serum (FBS), Dulbecco’s modified Eagle’s medium (DMEM) and 4′,6-diamidino-2-phenylindole (DAPI), were purchased from Gibco, Thermo Fisher Scientific (Waltham, MA, USA). Dimethyl sulfoxide (DMSO), 3-(4,5-2-yl)-2-5-diphenyl tetrazolium bromide (MTT), 5,5′,6,6′-tetrachloro-1,1′,3,3′-tetraethyl-imidacarbocyanine iodide (JC-1), 2,7-dichlorodihydrofluorescin diacetate (H2DCFDA), potassium persulfate (di-potassium peroxdisulfate), phosphate buffered saline (PBS) tablets, fetal bovine serum, and trypsin were purchased from Sigma-Aldrich (Saint Louis, MO, USA). Commercially available reagents for the determination of clotting times (Dia-PT and DiaPTT) were purchased from Diagon (Budapest, Hungary). The thrombin enzyme was provided by Biomed (Lublin, Poland) and 3 and Chromogenix S-2238^®^ substrate (H-D-Phenylalanyl-L-pipecolyl-Larginine-p-nitroaniline dihydrochloride) by Diapharma (Beckett Ridge, OH, USA). Blood from healthy donors and fresh human blood plasma for hemostatic assays derived from buffy coats (from healthy volunteers) were obtained from the Regional Blood Donation and Blood Treatment Center in Lodz (Poland). Membrane culture inserts for 24-well plates, PET, and 8 µM pores to check cell migration were purchased from Biokom (Janki, Poland). All other chemicals used were of analytical grade, and solutions were prepared using water purified by the Mili-Q system.

### 3.5. Hemolysis Assay

The red blood cells (RBCs) were collected after centrifugation of whole blood at 3000 rpm (10 min, 4 °C), and purified by three cycles of washing with PBS (phosphate buffered saline; pH = 7.4). To study the effect of alginate composites on erythrocyte stability, red blood cells (RBCs) suspended in PBS to a final hematocrit (HTC) of 10% were treated with different modified alginate composites, in the form of squares with dimensions 0.5 cm × 0.5 cm for 1 or 3 h. Erythrocytes in PBS (without alginate composite) were used as a control. After incubation, samples were centrifuged (3000 rpm, 10 min) and the absorbance of supernatant was measured spectrophotometrically (Jasco V-650, Jasco International Co., Osaka, Japan) at 540 nm to calculate the percentage of hemolysis according to the following equation:

% Hemolysis = As/Ac × 100%

where:

As represents the absorbance of the sample
Ac represents the absorbance of the erythrocytes in water (100% of hemolysis).

### 3.6. The Adsorption of Hemoglobin (Hb)

The experiment was carried out to investigate whether alginate composites adsorb hemoglobin from the environment. Alginate squares with dimensions of 0.5 cm × 0.5 cm were added to hemoglobin solutions (0.1% *v*/*v*). Samples were incubated at 37 °C for 1 or 3 h. After this time, the samples were centrifuged, and the absorbance of hemoglobin was measured at a wavelength of 540 nm. The percentage of hemoglobin adsorption was calculated from the formula:

Adsorption of Hb [%] = 100% − (As/Ac × 100%)

where:

As is the absorbance of the sample containing the alginate composites
Ac is the absorbance of the control (hemoglobin without alginate composites).

### 3.7. Methemoglobin

The concentration of hemoglobin was measured by the Drabkin method. Absorption spectra of hemoglobin were obtained for the wavelengths ranging from 440 to 700 nm using a spectrophotometer (Jasco V-650, Jasco International Co., Osaka, Japan) connected to a computer. The percentage of met-Hb in the total Hb content, after incubation with composites (1 or 3 h), was calculated from the absorbance at 630 and 700 nm. Hemoglobin treated with potassium ferricyanide (100% metHb) was used as a positive control.

% of met-Hb = [(A_630_ − A_700_)/(A_630*_ − A_700*_)] × 100%

where:

A_630_—the absorbance of a control sample and sample with composites at 630 nm,
A_700_—the absorbance of a control sample and sample with composites at 700 nm,
A_630*_—the absorbance of a control sample and sample with composites treated with potassium ferricyanide—100% met-Hb at 630 nm,
A_700*_—the absorbance of a control sample and sample with composites treated with potassium ferricyanide—100% met-Hb at 700 nm.

### 3.8. Cell Culture

BJ cells were grown as a monolayer in DMEM medium supplemented with 10% fetal bovine serum (FBS) and 1% streptomycin. The cultures were incubated at 37 °C in an atmosphere of 5% CO_2_. KERTr cells were grown in Keratinocyte Serum Free Medium (Gibco 1705-042^®^) with added Keratinocytes Supplements (Gibco 3700-015^®^) including Bovine Pituitary Extract (BPE, Gibco 13028-014^®^) and human recombinant epidermal growth factor (EGF, Gibco 10450-013^®^) further supplemented with additional 30 ng/ml human recombinated epidermal growth factor (EGF), was purchased from Gibco, Thermo Fisher Scientific (Waltham, MA, USA). The cultures were incubated at 37 °C in an atmosphere of 5% CO_2_. Both cell lines were split for subcultures every 2 days and were kept in log phase by regular passage according to the procedure described previously [56].

### 3.9. Cytotoxicity Assay

Cell viability was determined by the MTT assay after incubation (3 h) with alginate composites. In brief, BJ cells (5 × 104 cells/well) and KERTr cells (5 × 10^5^ cells/well) were seeded in a 24-well plate and kept overnight in the incubator (37 °C, 5% CO_2_). The next day, the medium was replaced with fresh medium and the alginate nanocomposite was added. After 6 3 h of incubation, cells were washed in PBS and incubated with 0.5 mg/mL of 3-(4,5- dimethylthiazol-2-yl)-2,5-diphenyl tetrazolium bromide (MTT) at 37 °C for 3 h. Then, the MTT was discarded carefully, and dimethyl sulfoxide (DMSO) was added to solubilize the formazan crystals [57]. Finally, the absorbance was measured for each well at a wavelength of 570 nm using a Bio-Tek Synergy HT Microplate Reader (Bio-Tek Instruments, Winooski, VT, USA). All experiments were performed in six repetitions. Cell viability was calculated as the percent ratio of absorbance of the samples to the reference control, according to the formula:

% Viability= As/Ac × 100%

where: As is the absorbance of the sample and Ac is the absorbance of the control (untreated cells).

### 3.10. Measurement of Reactive Oxygen Species (ROS)

To measure changes in the level of reactive oxygen species (ROS) in cells the H2DCFDA fluorescent probe (2′,7′-dichlorodihydrofluorescein diacetate) was used. H2DCFDA after deacetylation to DCFH2 (2′,7′-dichlorodihydrofluorescein) is oxidized intracellularly emitting fluorescence 2′,7′-dichlorofluorescein (DCF). The fluorescence intensity increases linearly with an increase in the amount of reactive oxygen species in the cell [58]. BJ cells were seeded in black 96-well plates at 1.25 × 10^4^ per well while KERTr cells at 2.5 × 10^4^ per well. After 3 h of incubation with the materials, the medium was removed, and the cells were washed with PBS before 50 µL 2 μM H2DCFDA was added. The probe plate incubation time was 15 min. The solution was recovered, and 50 µL of PBS was added per well. Samples were analyzed using a Fluoroscan Ascent FL microplate reader (BioTek, Synergy HTX multi-mode reader, Winooski, VT, USA) with an excitation wavelength of λex = 495 and emission wavelength of λem = 529.

### 3.11. Assessment of Mitochondrial Membrane Potential (ΔΨm)

The mitochondrial membrane potential (ΔΨm) was estimated using fluorescent probe JC-1 (5,5′,6,6′-tetrachloro-1,1′,3,3′-tetraethylbenzimidazolylcarbocyanine iodide) [59]. Cells were seeded into black 96-well tissue culture plates with a transparent bottom (Greiner) at a density of 5 × 10^4^ cells/well (BJ) or 5 × 10^5^ cells/well (KERTr) in a 100 µL culture medium and allowed to adhere overnight, and then treated with alginate composites for 3 h. Finally, the cells were preincubated with 5 µM JC-1 in CO2 incubator at 37 °C for 30 min. The fluorescence was measured on a Bio-Tek Synergy HT Microplate Reader (Bio-Tek Instruments, Winooski, VT, USA). Results are shown as a ratio of fluorescence of dimers (measured at 530/590 nm) to monomers (measured at 485/538 nm). All compounds were tested in six duplicates and ΔΨm was calculated from the formula:

ΔΨm = Fd/Fm

where:

ΔΨm—mitochondrial transmembrane potential directly proportional to the fluorescence coefficient,
Fd—dimer fluorescence,
Fm—monomer fluorescence.

### 3.12. Genotoxicity

The comet assay was performed at pH > 13 essentially according to the published procedure [60]. The experiment assay was modified as described by Blasiak and Kowalik [61]. A freshly prepared suspension of the cells (5 × 10^4^ cells/mL) in 0.75% LMP agarose dissolved in PBS was spread onto microscope slides precoated with 0.5% NMP agarose. The cells were lysed for 1 h at 4 °C in a buffer consisting of 2.5-M NaCl, 100-mM EDTA, 1% Triton X-100, 10-mM Tris, and pH 10. After the lysis, the slides were placed in an electrophoretic buffer (300 mM NaOH, 1 mM EDTA, pH > 13) for 20 min to allow the unwinding of DNA. Electrophoresis was conducted in the same buffer at temperature of 4 °C for 20 min at an electric field strength of 0.73 V/cm (28 mA). The slides were then washed in water, drained and stained with 2-mg/ml DAPI, and covered with cover slips. To prevent additional DNA damage, all the steps described above were conducted under dimmed light or in the dark. Each experiment in the alkaline version of the comet assay included a positive control, which were both lines cells incubated with H_2_O_2_ at 20 mM for 10 min at 4 °C. The comets were observed at 200 magnifications in an Eclipse fluorescence microscope (Nikon, Tokyo, Japan) attached to COHU 4910 video camera (Cohu, San Diego, CA, USA) equipped with a UV-1 filter block (an excitation filter of 359 nm and a barrier filter of 461 nm) and connected to a personal computer-based image analysis system LuciaComet v. 6.0 (Laboratory Imaging, Praha, Czech Republic). Fifty images were randomly selected from each sample.

### 3.13. Cell Migration

BJ and KERTr cells migration was assessed according to the procedure described previously [56]. Both cell lines were starved overnight in serum-free medium with 0.2% bovine serum albumin (BSA). Cells were prepared in a concentration of 2.5 × 10^5^ cells/mL. ThinCert™ cell culture inserts were placed in a multiwell cell culture plate, thereby forming two compartments: the upper compartment of the insert and the lower compartment of the plate well. Both compartments formed the migration chamber, separated by the porous PET membrane. The plate with cells with alginate composites was incubated for 24 h in an incubator at 37 °C and 5% CO_2_ in air. Finally, the viability of the migrating cells was determined using the MTT test and measured spectrophotometrically at 570 nm.

### 3.14. Measurements of Prothrombin Time (PT)

Prothrombin time (PT) was determined coagulometrically using an Optical Caogulometer K-3002 (Kselmed, Grudziadz, Poland), according to the protocol provided by the manufacturer. Briefly, after 15 min of pre-incubation with alginate composites, the human plasma (50 μL) was incubated for 2 min at 37 °C in the analyzer thermoblock and then, directly before measurement, 100 μL of Dia-PT liquid (commercial preparation: Diagon, Budapest, Hungary) was added.

### 3.15. Measurements of Thrombin Time (TT)

Thrombin time (TT) was determined coagulometrically using an Optical Caogulometer K-3002 (Kselmed, Grudziadz, Poland), according to the protocol provided by the manufacturer. Briefly, after 15 min of pre-incubation with alginate nanocomposites, the human plasma (50 μL) was incubated for 2 min at 37 °C and then, directly before measurement, 100 μL of thrombin (Biomed, Lublin, Poland) was added (at the final concentration of 5 U/mL).

### 3.16. Amidolytic Activity of Thrombin

Thrombin (Biomed, Lublin, Poland; 0.75 U/ml, in the tris-buffered saline/TBS, pH 7.4) was pre-incubated with alginate nanocomposites for 15 min, at 37 °C. The reaction mixture contained 40 µL of 3 mM Chromogenix S-2238^®^ substrate (H-D-Phenylalanyl-L-pipecolylLarginine-p-nitroaniline dihydrochloride; Diapharma, Beckett Ridge, OH, USA) and 280 µL of the thrombin solution, i.e., the control/untreated thrombin or enzyme pre-incubated with the examined alginate composites. Measurements were executed in 96-well microplates, using a kinetic mode of the BMG Labtech Spectrostar Nano microplate spectrophotometer. Absorbance changes were recorded every 15 s for 10 min, at 415 nm. The hydrolytic activity of thrombin in all samples was estimated based on the maximal velocity of the reaction (Vmax).

### 3.17. Statistical Analysis

Data are presented as the mean ± SD from minimum of 3 sets of measurements. Statistical differences between the control and treatment groups were analyzed by one-way ANOVA followed by Tukey’ analysis. *p* < 0.05 was taken as statistically significant.

## 4. Conclusions

Alginate is known both in the literature and even commercially as a promising scaffold for wound dressings. Surprisingly, despite the interest of downsizing metal oxide for biological purposes, few studies have focused on merging alginate and glassy-like metal oxide phase to generate homogenous nanocomposite films. Therefore, we have attempted to embed glassy-like metal oxide inside of the corresponding alginate films and investigated the possible use of the final composites in wound healing. Different films were prepared including alginate-titanium dioxide, alginate-germanium oxide, alginate-iron oxide, alginate-vanadium oxide and alginate-zinc oxide. We have moreover succeeded in varying the amount of the metal oxide grown inside without compromising the flexibility of the films or their transparency. We have also shown that dissolution of these films provides water-soluble nanoparticles and the alginate shell prevents them from sedimentation.

Among the analyzed alginate composites modified with metal oxides, the most detrimental was the one functionalized by vanadium and iron oxide species. The most promising was the alginate-titanium oxide composite (Alg@(Ti-O-Ti)_n_), which showed low cytotoxicity and genotoxicity towards fibroblasts and keratinocytes. It did not cause a significant decrease in the cellular potential and generation of reactive oxygen species. Moreover, it showed the greatest stimulating effect on cell migration in both cell lines, which plays an important role in the healing process. Studies on human blood erythrocytes proved that Alg@(Ti-O-Ti)_n_ does not show significant hemolytic properties. It also acts as a procoagulant during the prothrombin time. The obtained results complement and extend the current state of knowledge on alginate materials in wound dressings.

## Data Availability

Not applicable.

## Supplementary Materials

The following supporting information can be downloaded at: https://www.mdpi.com/article/10.3390/ijms23105585/s1.
