# Supplementary material for: Glassy-like Metal Oxide Particles Embedded on Micrometer Thicker Alginate Films as Promising Wound Healing Nanomaterials
Source: Int J Mol Sci. 2022 May 17;23(10):5585. doi: 10.3390/ijms23105585 (PMC9142123; doi:10.3390/ijms23105585)
Supplement: Supplementary file 1 [file ijms-23-05585-s001.zip › ijms-1713926-supplementary.pdf]

Glassy-Like Metal Oxide Particles Embedded on Micrometer Thicker Alginate Films as Promising Wound Healing Nanomaterials

Marta Kedzierska,[1,*] Nisrine Hammi,[2] Joanna Kolodziejczyk-Czepas,[3] Nadia Katir,[2] Maria Bryszewska,[1] Katarzyna Milowska,[1] and Abdelkrim El Kadib,[2,*]

[1] Department of General Biophysics, Faculty of Biology and Environmental Protection, University of Lodz, Pomorska 141/143, 90-236 Lodz, Poland.; marta.kedzierska@biol.uni.lodz.pl (M.K.); maria.bryszewska@biol.uni.lodz.pl (M.B.); katarzyna.milowska@biol.uni.lodz.pl (K.M.)
[2] Euromed Research Center, Engineering Division, Euro-Med University of Fes (UEMF), Route de Meknes, Rond-point de Bensouda, 30070, Fès, Morocco.; n.hammi@ueuromed.org (N.H.); n.katir@ueuromed.org (N.K.); a.elkadib@ueuromed.org (A.E.K.)
[3] Department of General Biochemistry, Faculty of Biology and Environmental Protection, University of Lodz, Pomorska 141/143, 90-236 Lodz, Poland.; joanna.kolodziejczyk@biol.uni.lodz.pl (J.K.-C.)

* Correspondence: marta.kedzierska@biol.uni.lodz.pl; a.elkadib@ueuromed.org

Contents

S1. Experimental section 2
S2. SEM and EDX analysis of alginate-metal oxide films 3
S3: DRIFT spectra obtained for alginate-metal oxide films 5
S4: XRD results obtained for alginate-metal oxide films 6
S5: TGA results obtained for alginate-metal oxide films 7
S6: TEM analysis of alginate-metal oxide solution 8

S1. Experimental section

Table S1a: Preparation of Alginate-metal oxide films

Sample	Metal precursors	r_T [a]	C (g/L)[b]	TGA residue (%)	r_E [c]
Alg@(Ti-O-Ti)$_n$	$Ti(acac)_2OiPr_2$	1:1	40	28	1:1.05
Alg@(V-O-V)$_n$	$V(acac)_3$	1:1	40	28	1 :1.01
Alg@GeO_2	$Ge(OEt)_4$	1:1	29	34	1 :1.06
Alg@ZnO-5:1	Zn $(OAc)_2$	5:1	4	-	-
Alg@ZnO-10:1	Zn $(OAc)_2$	10:1	2.1	-	-
Alg@ZnO-20:1	Zn $(OAc)_2$	20:1	1.05	-	-
Alg@ZnO(Cl)-5:1	$ZnCl_2$	5:1	3.1	-	-
Alg@ZnO(Cl)-10:1	$ZnCl_2$	10:1	1.5	-	-
Alg@ZnO(Cl)-20:1	$ZnCl_2$	20:1	0.7	-	-
Alg@(Fe-O-Fe)$_n$	$Fe(acac)_3$	1:1	40	27	1 :1.05

[a]: r_T, the theoretical molar ratio of COO-:metal precursor; [b]: C, Concentration of the alcoholic solution of the metal alkoxide precursor (g/L); [c]: r_E, the experimental molar ratio of COO-:metal precursor. The calculation of the molar ratio of Alg@(Ti-O-Ti)$_n$, Alg@(V-O-V)$_n$, Alg@GeO_2 and Alg@(Fe-O-Fe)$_n$ was based on the mass residue of TiO_2, VO_2, GeO_2 and Fe_2O_3, respectively, analyzed via TGA under air.

Table S1b: Water-soluble chitosan- coated –metal oxide nanoparticles

7 mg of Alg@(M-O-M)n films were immersed in aqueous solution and kept under stirring for different periods of time.

Films	Mass (mg)	Solvent	Volume (mL)	Dissolution time (min)
Alg	7	Water	3	<1
Alg@(Ti-O-Ti)$_n$-1:1	7		3	30
Alg@(V-O-V)$_n$-1:1	7		3	4
Alg@GeO_2-1:1	7		3	2
Alg@(Fe-O-Fe)$_n$-1:1	7		3	4

S2. SEM and EDX analysis of alginate-metal oxide films

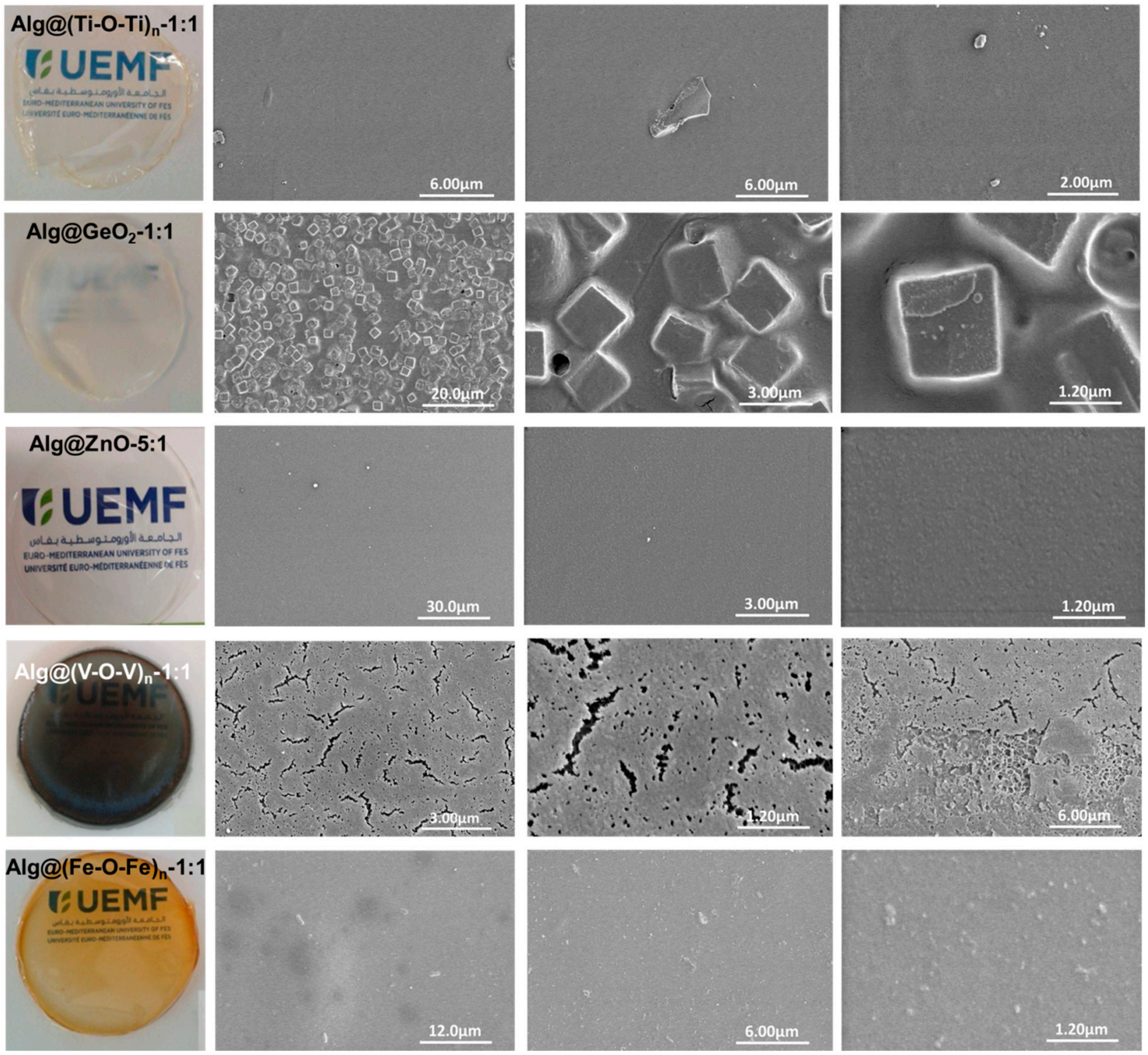

Figure S2a. SEM images obtained for Alg@(M-O-M)$_n$ films

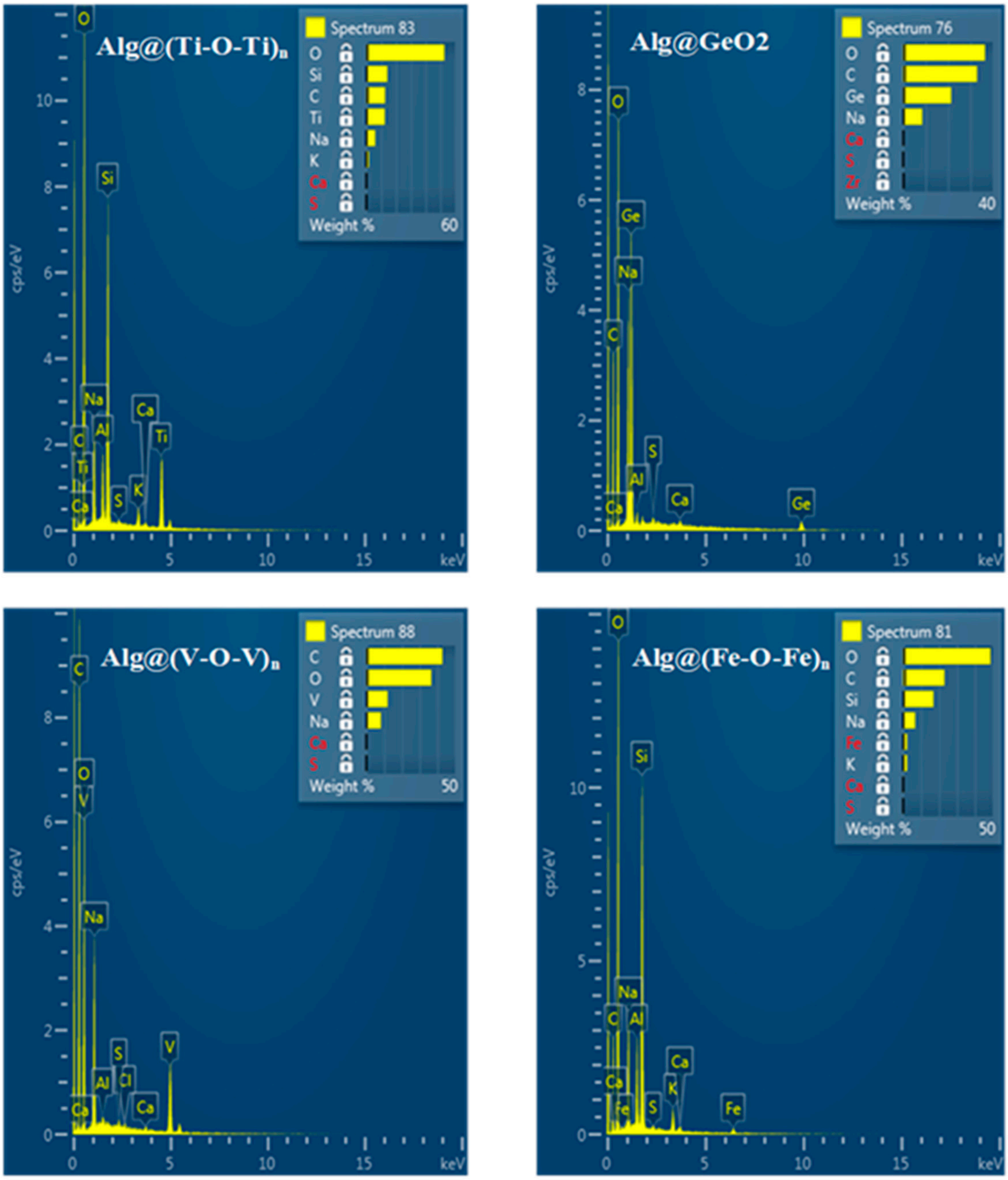

Figure S2b. EDX analysis of Alg@(M-O-M)n films

S3: DRIFT spectra obtained for alginate-metal oxide films

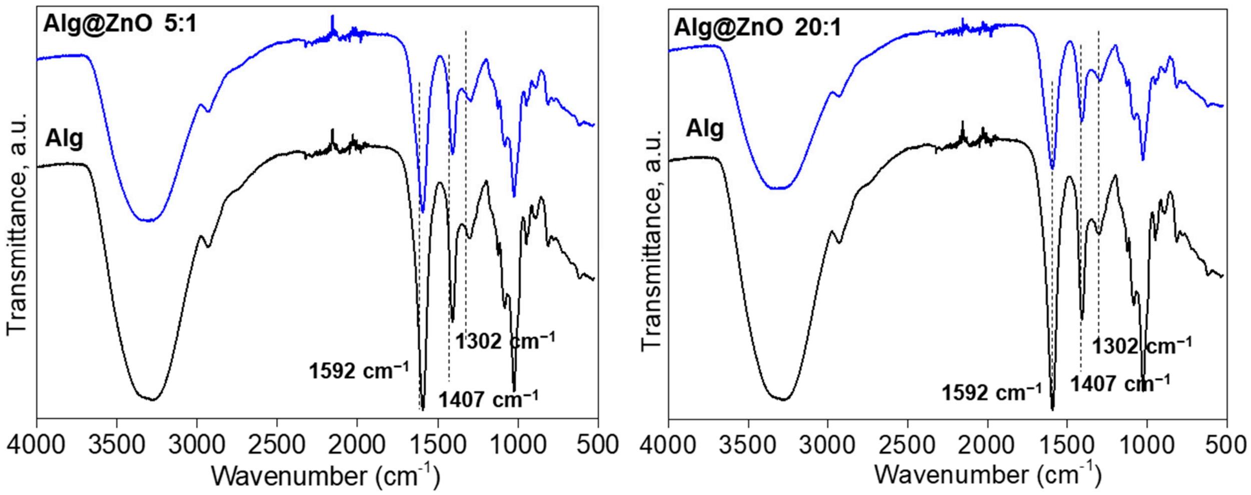

Figure S3. DRIFT spectra obtained for Alg@ZnO films

S4: XRD results obtained for alginate-metal oxide films

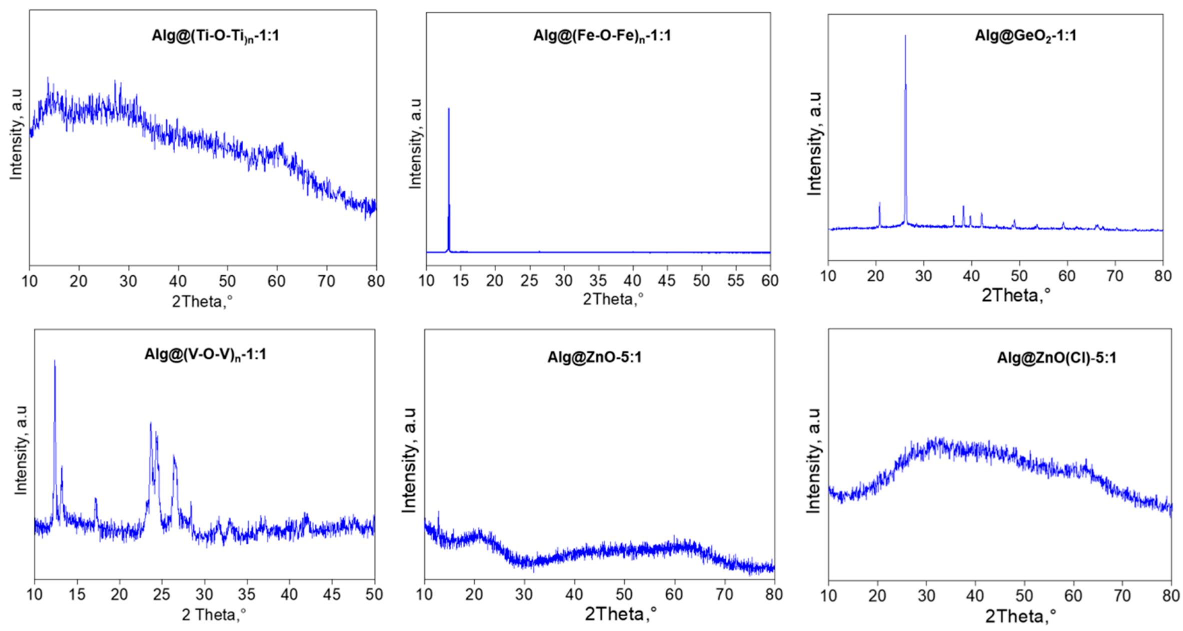

Figure S4. X- ray diffractogramms obtained for Alg@(M-O-M)n films

S5: TGA results obtained for alginate-metal oxide films

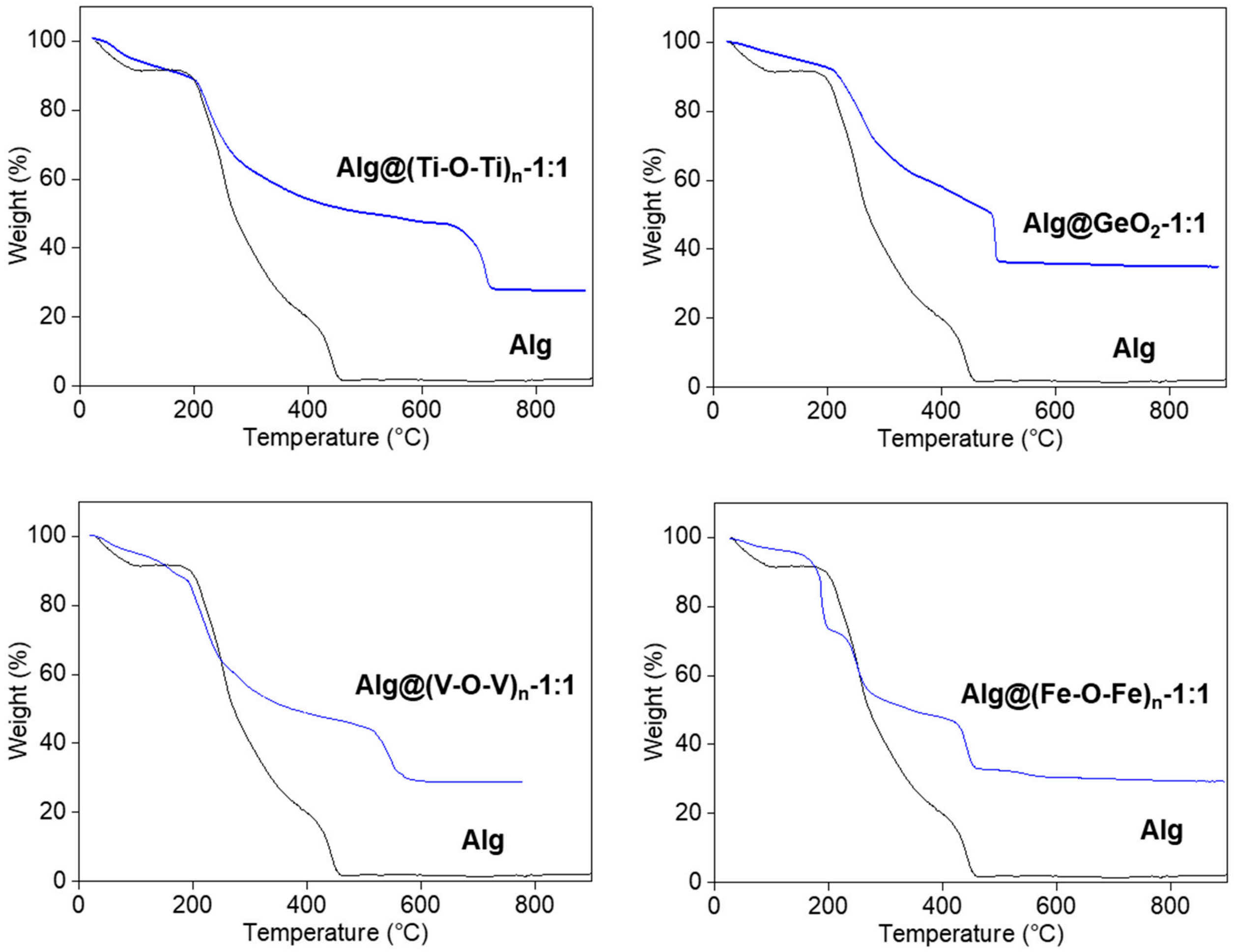

Figure S5. TGA results obtained for alginate-metal oxide Alg@(M-O-M)n films

S6: TEM analysis of alginate-metal oxide solution

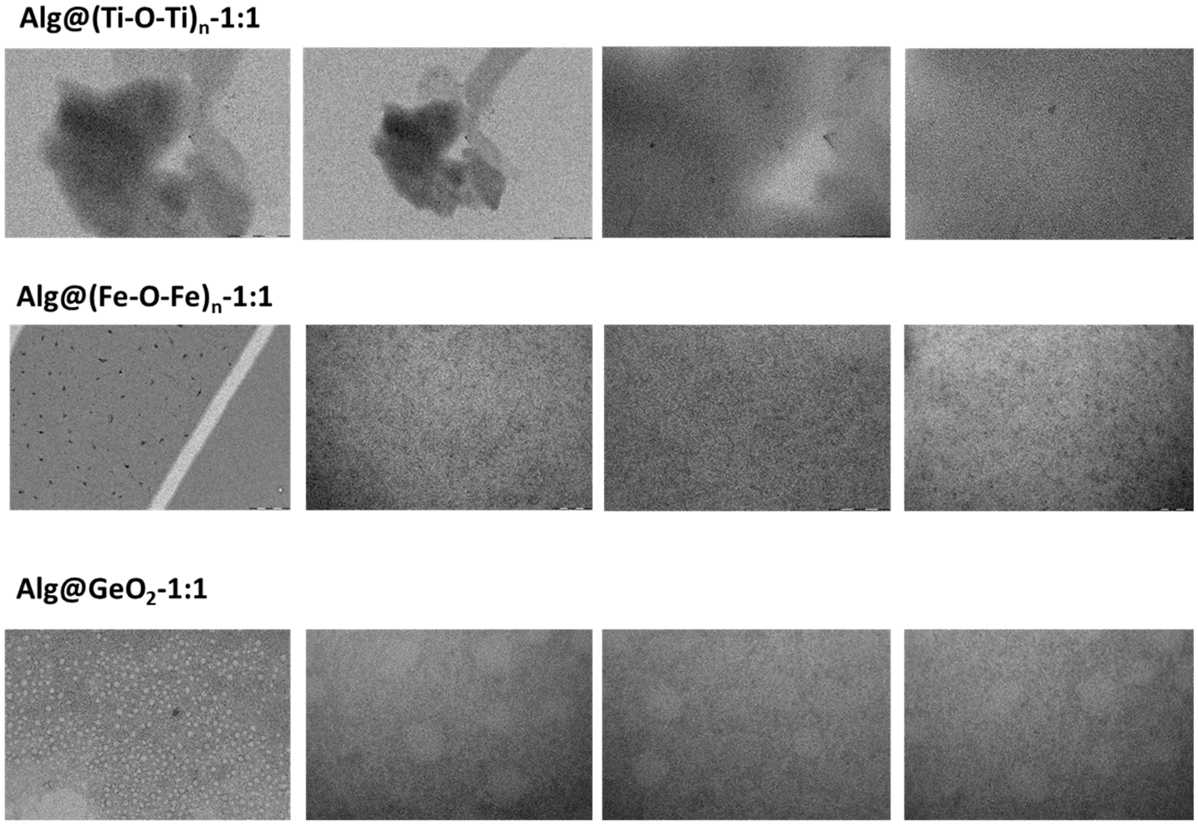

Figure S6. TEM images obtained for Alg@$(M\text{-}O\text{-}M)_n$ solutions
